# Diagnosis of Pediatric Nasopharynx Carcinoma after Recurrent Adenoidectomy

**DOI:** 10.1155/2013/653963

**Published:** 2013-12-11

**Authors:** Koray Cengiz, Tolgar Lütfi Kumral, Güven Yıldırım

**Affiliations:** ^1^Department of Otorhinolaryngology-Head and Neck Surgery, Private Gazi Hospital, Sultangazi, Istanbul 34260, Turkey; ^2^Department of Otorhinolaryngology-Head and Neck Surgery, Okmeydanı Training and Research Hospital, Okmeydanı, Şişli, Istanbul 34400, Turkey

## Abstract

Nasopharyngeal soft tissue is most commonly adenoid hypertrophy in children. Although rare, nasopharyngeal carcinoma (NPC) does occur in children. Nasal obstruction, serous otitis media, hearing problems, and tinnitus are common signs and symptoms of all nasal diseases. For this reason, the majority of NPC presents with advanced disease at the time of the diagnosis. This paper reported 7-year-old boy who was admitted to the hospital for adenoidectomy. He had recurrent adenoidectomy operation due to nasal obstruction. NPC had been diagnosed suspecting the hard mass in the nasopharynx during the operation. Adenoidectomy is the most commonly performed surgical procedure in ENT practice and NPC is unlikely to be considered in the differential diagnosis. Surgeon should be careful about signs and symptoms that alert suspicion.

## 1. Introduction

Nasopharyngeal soft tissue is most commonly adenoid hypertrophy in children. However, NPC is rare accounting for 1–3% of all pediatric malignancies and pediatric NPC constitutes 20–50% of all NPC [1, 2]. The annual incidence of NPC is 0.3 per million at age 0–14 years, and 1 to 2 per million at age 15–19 years [3]. Peak is the fifth and sixth decades.

According to WHO classification, NPC is histopathologically divided into three categories: keratinizing squamous cell carcinoma (WHO type I), nonkeratinizing squamous cell carcinoma (WHO type II), and undifferentiated carcinoma (WHO type III). Most of the cases are diagnosed as undifferentiated nasopharynx carcinoma (WHO type 3) in the advanced stage [4].

Epstein-Barr virus (EBV) infection combined with frequent exposure to environmental carcinogens is suggested in the etiology [5]. Five-year disease-free survival rates are between 29–60% [6].

Radiation therapy is effective in 75–100% of local control of the locoregional disease. Systemic metastasis at diagnosis is between 30–50% and this creates the main difficulty in the treatment.

This study presents a case of childhood NPC suspected during adenoidectomy. This is a very common surgical procedure in daily practice and surgeons should keep this diagnosis in their minds. This study also reveals alarming signs and symptoms for diagnosis.

## 2. Case Report

Seven-year-old male patient was admitted with complaints of nasal obstruction and snoring. He had a complaint of nosebleeds for the last two days. ENT examination was unremarkable. Flexible nasal endoscopy showed a soft tissue mass compatible with adenoid hypertrophy in the choana. Lateral radiograph of the nasopharynx revealed obstruction in the nasopharynx, so adenoidectomy operation was planned. The patient had previous adenotonsillectomy and adenoidectomy, respectively, at the ages of 2.5 years and 5 years old without any pathological examination. Nasal congestion and obstruction were relieved postoperatively despite recurrence over time. Otoscopic examination was normal without effusion and there was multiple palpable lymphadenopathy less than 2 cm in the neck.

Lateral pharyngeal bands were fibrotic and tight during surgery and there was a hard, dense mass in the nasopharynx. As the mass was harder than expected adenoid tissue, it was sent to postoperative pathology. After the immunohistochemical staining in the paraffin blog specimen, pathology has been reported compatible with undifferentiated NPC showing marked pleomorphism and hyperchromatic nuclei with atypical epithelial cells. The specimens were stained pancytokeratin (+), CD 3, and CD 20 (−).

**Figure 1:**
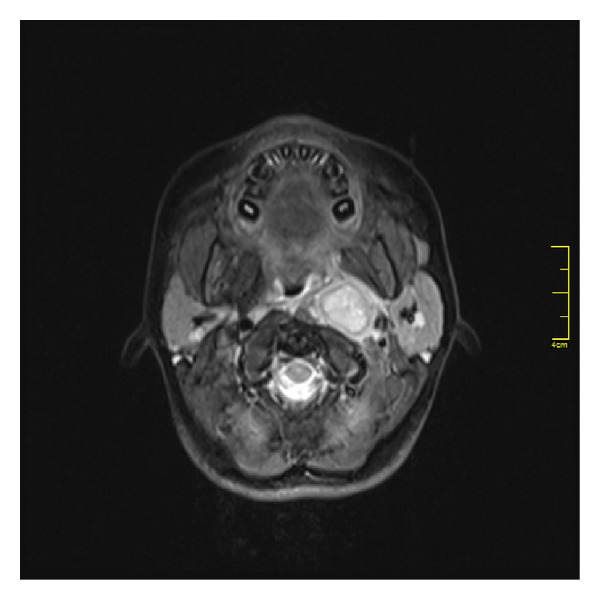
In the contrasted neck MRI, there was 3 × 3 cm axial size, hyperintense in T2 weighted, left parapharyngeal space occupying lesion on the left infratemporal fossa adjacent to the medial-inferior border.

Contrasted neck MRI after the surgery revealed 3 × 3 cm axial size, left parapharyngeal space occupying lesion on the left infratemporal fossa adjacent to the medial-inferior border. The patient had bilateral, multiple, 2 × 2 cm maximum diameter lymphadenopathy, prominent in the left jugulodigastric region (Figure 1).

**Figure 2:**
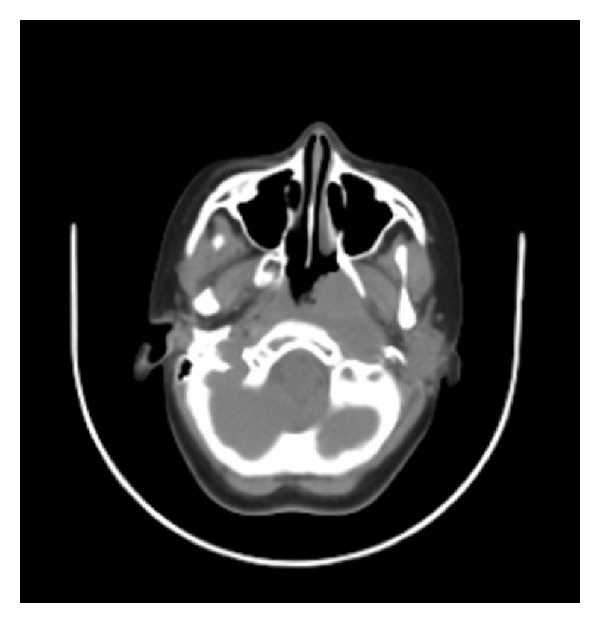
In the computerized tomography, there was a 37 × 30 mm size centrally hypometabolic, peripherally hypermetabolic mass starting from the base of the nasopharynx occupying the left Rosenmuller fossa. The mass obliterated the carotid space extending to the left parapharyngeal space.

In FDG PET, there was a 37 × 30 mm size centrally hypometabolic, peripherally hypermetabolic mass starting from the base of the nasopharynx occupying the left Rosenmuller fossa (Figure 2). The mass obliterated the carotid space extending to the left parapharyngeal space (SUV 4–6). There were also hypermetabolic lymphadenopathies in the left neck level II, III, and IV (SUV 4-5).

Tumor was extending to soft tissues of oropharynx and nasal fossa with parapharyngeal extension. There was 2 × 2 cm lymphadenopathy in the left upper jugulodigastric region. Tumor was evaluated as T2N1MO and Stage 2B.

The patient had 50 Gy radiotherapy and chemotherapy had been planned after radiotherapy. The patient is under the one-year follow-up and had no recurrence.

## 3. Discussion

Childhood NPC varies widely according to race and geography. At presentation, NPC associated with hearing problems, serous otitis media, tinnitus, nasal obstruction, anosmia, bleeding, difficulty in swallowing and dysphonia, and even eye symptoms with diplopia and pain. These findings are common signs and symptoms of all diseases of the nose. For this reason NPC usually presents with advanced locoregional disease [7]. The most common symptom is unilateral ear complaint which is also very insidious and cervical mass at the diagnosis. In this case report, the patient had two times previous adenoidectomy due to nose obstruction and it was suspected at the third time. Late diagnosis in the NPC is because of ignoring or misdiagnosing the unspecific symptoms mimicking upper respiratory tract infection during early stages.

The flexible fibreoptic endoscope examination and biopsy are golden standard to establish the diagnosis. However, it is very difficult to apply to the pediatric age group in routine. Also Kalcioglu et al. reported that microscopic analysis of routine tonsillectomy and/or adenoidectomy specimens may not be necessary unless clinically suspicious [8].

Most of the time, NPC is unlikely to be considered in the differential diagnosis of the adenoid hypertrophy. Rhabdomyosarcoma or lymphoma is the more likely diagnosis in younger children whereas NPC or lymphoma is more common in adolescents.

NPC initiates in the fossa of Rosenmuller and spreads intracranially or locally as a mass in the head. In our case, NPC was diagnosed suspecting the hard mass originating from the left Rosenmuller fossa. It can invade petroclival fissure, skull base, pterygopalatine fossa, and parapharyngeal space.

Configuration of the mass is always asymmetric in carcinoma, while adenoid hypertrophy is symmetric, diffuse mass without invasion of environmental structures. However, magnetic resonance imaging (MRI) cannot be applied routinely to all adenoidectomy planned patients. MRI can be applied to suspected cases particularly to pathological lymphadenopathy. In our case, MRI performed after nasopharyngeal carcinoma diagnosis (Figure 2).

The treatment of nasopharyngeal carcinoma in children is high-dose locoregional radiotherapy to the area as in adults. Several authors advocate the use of systemic adjuvant chemotherapy to reduce distant metastasis and combined modality therapy increases overall survival [9]. However optimal radiation dose and its sequencing with chemotherapy remain to be defined. In this case, the patient had 50 Gy radiotherapy and there was no complication in one-year follow-up.

Early age EBV infection in nasopharyngeal epithelial tissues due to locoregional inflammation may cause NPC development. Transformation of epithelial cells into a malignant disease and premalignant dysplasia may progress rapidly into cancer. Lo et al. showed that EBV DNA was detectable in the plasma samples and can be used for screening [10].

Due to the late signs and symptoms and difficulties in diagnosis and biopsy (such as nasopharyngeal brushing and blood investigations and adjuvant laboratory examinations), screening tests are of utmost importance for early diagnosis [11].

EBV viral capsid antigen and EBV DNA and EBVIgA serology may be used for early identification of individuals at risk for screening programs. EBER hybridization (EBERRISH) is considered the gold standard for detecting and localizing latent EBV in tissue specimens, whether frozen or formalin fixed and paraffin embedded [12]. But it is expensive and needs to be developed.

Early stage NPC is difficult to diagnose clinically because of its hidden localization in the nasopharynx. Although rare, nasopharyngeal carcinoma does occur in children and surgeon should be careful about this. Surgeons should always keep in their minds NPC in pediatric adenoid vegetation. The physician should be aware of early warning signs.
